# Change in skeletal muscle stiffness after running competition is dependent on both running distance and recovery time: a pilot study

**DOI:** 10.7717/peerj.4469

**Published:** 2018-03-12

**Authors:** Seyedali Sadeghi, Cassidy Newman, Daniel H. Cortes

**Affiliations:** 1 Department of Mechanical and Nuclear Engineering, Pennsylvania State University, State College, PA, United States of America; 2 Department of Biomedical Engineering, Pennsylvania State University, State College, PA, United States of America

**Keywords:** Running distance, Shear wave speed, Muscle mechanical properties, Shear wave elastography, Running time

## Abstract

Long-distance running competitions impose a large amount of mechanical loading and strain leading to muscle edema and delayed onset muscle soreness (DOMS). Damage to various muscle fibers, metabolic impairments and fatigue have been linked to explain how DOMS impairs muscle function. Disruptions of muscle fiber during DOMS exacerbated by exercise have been shown to change muscle mechanical properties. The objective of this study is to quantify changes in mechanical properties of different muscles in the thigh and lower leg as function of running distance and time after competition. A custom implementation of Focused Comb-Push Ultrasound Shear Elastography (F-CUSE) method was used to evaluate shear modulus in runners before and after a race. Twenty-two healthy individuals (age: 23 ± 5 years) were recruited using convenience sampling and split into three race categories: short distance (nine subjects, 3–5 miles), middle distance (10 subjects, 10–13 miles), and long distance (three subjects, 26+ miles). Shear Wave Elastography (SWE) measurements were taken on both legs of each subject on the rectus femoris (RF), vastus lateralis (VL), vastus medialis (VM), soleus, lateral gastrocnemius (LG), medial gastrocnemius (MG), biceps femoris (BF) and semitendinosus (ST) muscles. For statistical analyses, a linear mixed model was used, with recovery time and running distance as fixed variables, while shear modulus was used as the dependent variable. Recovery time had a significant effect on the soleus (*p* = 0.05), while running distance had considerable effect on the biceps femoris (*p* = 0.02), vastus lateralis (*p* < 0.01) and semitendinosus muscles (*p* = 0.02). Sixty-seven percent of muscles exhibited a decreasing stiffness trend from before competition to immediately after competition. The preliminary results suggest that SWE could potentially be used to quantify changes of muscle mechanical properties as a way for measuring recovery procedures for runners.

## Introduction

Long-distance running competitions have become increasingly popular in the last decade. However, these competitions impose a large amount of mechanical loading and strain to the leg caused by repetitive muscle contraction. As a result of increase in internal shear strain during mechanical loading, cytoskeletal inside tissue will be altered inducing neuromuscular functional impairments and muscle-fiber damage, muscle edema, and delayed onset muscle soreness (DOMS) (Pearcey et al., 2015). Disrupted muscle fibers cause an increase in lipid content and change in muscle mechanical properties (Proske & Morgan, 2001). Quantifying mechanical properties non-invasively after training and competitions can potentially provide information about microstructural changes in the muscle. This will improve our understanding of the relationships between exercise intensity, symptoms (tightness, weakness or sharp pain in lower leg muscles), muscle remodeling and injury in runners. Muscle mechanical properties can potentially be used as biomarkers for muscle health. Shear Wave Elastography (SWE) is an ultrasound method that has been used to measure the shear modulus in soft tissues such as skeletal muscles (Bercoff, Tanter & Fink, 2004; Cortes et al., 2015; Palmeri & Nightingale, 2011). Compared to other imaging techniques such as magnetic resonance imaging, SWE is relatively faster, cheaper and capable of assessing the morphological and mechanical properties of the muscle at the same time. However, there is limited information on the change of shear modulus in leg muscles as function of running distance and recovery after competition.

Changes in muscle mechanical properties, associated with performance and injury, may be related to the increased joint stiffness typically observed after prolonged physical activity. In terms of performance, it was found that increased stiffness is associated with increased jump velocity, jump height and running economy (measured by oxygen consumption). Regarding injury, although there are no prospective studies that directly correlate injury and stiffness, some retrospective studies suggest that too much stiffness may be related to the bony injuries, while too little stiffness may result in soft tissue injuries (Brazier et al., 2014; Butler, Crowell & Davis, 2003; Kalkhoven & Watsford, 2017). Bobbert, Hollander & Huijing (1986) quantified Electromyography (EMG) and volume change on both legs in a healthy athletic group during DOMS. No change was observed between resting EMG levels of two legs over the pre-exercise level when soreness was present, not even 48 h post exercise, although at that time the sore area of the muscle was located very close to the recording site of electrodes. However, they found a rise in volume of the leg performing exercise compared to the control leg 24, 48 and 72 h post exercise, in which the swelling was indirectly linked to the increased muscle passive torque (Howell, Chleboun & Conatser, 1993). Jones, Newham & Clarkson (1987) assessed the force required to extend the elbow following DOMS-producing eccentric exercise and reported that such force increased with a delayed time after exercise. Chleboun et al. (1998) found that muscle swelling does not necessarily account for the sudden increase in post exercise stiffness, but may play a role in the subsequent muscle stiffness afterward. These studies suggest that muscle mechanical properties and resting tone may change after DOMS-inducing exercise.

SWE is an imaging technique that has been applied to measure mechanical properties (e.g., shear modulus) in muscles. SWE has been shown to be a reliable method when the transducer is placed parallel to the muscle fiber orientation (Alfuraih et al., 2017; Song et al., 2013). Saeki et al. (2017) investigated the reliability of shear elastic modulus measurement for the ankle plantar flexion muscles at different ankle postures. Their results showed that the interday reliability of the measurements is dependent to the ankle positions, reporting the maximum reliability at 20° dorsiflexion. The results showed that the CV for the lateral gastrocnemius (LG), medial gastrocnemius (MG) and Soleus were 4.7 to 6.7%, 5.7 to 6.5% and 9.4 to 12.0%, respectively. Brandenburg et al. (2015) evaluated the reliability of SWE when measuring passive bilateral lateral gastrocnemius muscle stiffness in children. The reliability of measurements were good to excellent (mean [95% confidence interval] range of reliability, 0.67 [0.44–0.83] to 0.80 [0.63–0.90]) when probe was placed on the skin with minimal pressure. MacDonald et al. (2016) assessed the intra-day and inter-rater reliability of shear modulus measured in abdominal muscles during trunk stability exercises, reporting shear modulus measurements were more reliable for superficial muscles than for deeper muscles. However, Dubois et al. (2015) demonstrated SWE as a reliable method for evaluating shear modulus of lower limbs, while probe positioning is not a critical factor for SWE reliability. SWE has been successfully applied to evaluate the mechanical properties of muscles and characterize their changes following injuries (Cortez et al., 2016; Eby et al., 2015; Yoshida et al., 2017). Andonian et al. (2016) employed SWE to assess the change in quadriceps stiffness before, throughout, and after an extreme mountain ultramarathon, reporting a decreasing trend during the race, followed by an increase in stiffness 48 h after the race. This preliminary finding shows that SWE has the potential to quantify muscle mechanical property changes after exercise, but has focused on one competition distance (Ryu & Jeong, 2017). However, running distance and recovery time may affect different leg muscles in different ways (particularly the ones subjected to more force, loading rate and shock).

Long-distance running induces numerous cellular changes in the structure and function of leg muscles depending on the running distances and recovery time. Examination of shear modulus of lower leg muscles in runners using SWE at different running distances at different time points before and after running may help evaluating physiological changes of lower leg muscles after running competitions. Therefore, SWE may determine whether obtained quantitative shear modulus can serve as reliable tool and useful biomarkers for monitoring changes in muscle stiffness under different extreme stresses. This information may provide insight for optimizing muscle stiffness and performance to reduce fatigue and muscle disorders in runners.

The objective of this study is to quantify changes in mechanical properties of different muscles in the thigh and lower leg as function of running distance (short-medium and long) and time after competition. We hypothesize that the shear modulus of muscle decreases shortly after competition and increases to pre-competition values after recovery. We further hypothesize that changes in shear modulus will be more pronounced after long-distance competitions since the inflammation process has shown to have greatest increase in long distance running, particularly after the second half of the race (Kim, Lee & Kim, 2007). Results from this study will provide the background information needed to design appropriate training strategies and recovery procedures for runners.

## Methods

### Shear wave elastography system

A Verasonic ultrasound system (Verasonic Inc., Redmond, WA, USA) with a L11-4V transducer (128 elements, beamwidth = 4–11 MHz, center frequency = 6.25 MHz, Philips Healthcare, Andover, MA, USA) was used in this study. A custom implementation of the Focused Comb-Push Ultrasound Shear Elastography (F-CUSE) introduced by Song et al. (Mehrmohammadi et al., 2015; Song et al., 2013; Song et al., 2012) was used to measure muscle shear modulus. The 64 transducer elements were divided into two subgroups which transmitted ultrasound push pulses simultaneously. The region of interest (ROI) size was adjusted to 7.39 mm * 7.39 mm with two focal points located at the middle of the right and left vertical line of the ROI. Each push pulse corresponded to a duration of 80 µs for 500 push cycles at 6.25 MHz frequency. Acoustic output intensity was measured to ensure the method satisfied FDA limits of intensity for use in human subjects. The propagation speed of shear wave was calculated within the ROI using a frame rate of 10,000/s. Finally, the corresponding shear modulus map could be constructed based on the obtained shear wave speed.

### Proposed protocol

The Institutional Review Board (IRB) of the Pennsylvania State University approved the study (STUDY00005680), and all subjects gave informed consent prior to any evaluation. The healthy volunteers were instructed about the objective and requirements of our study. Twenty-two subjects (age: 23 ±  5 years) were recruited using convenience sampling through the Penn State Club Cross Country Team and the Nittany Valley Running Club in State College, PA. Runners with a history of significant Achilles tendon pain, previous Achilles tendon surgery, or a history of systemic inflammatory disorders were excluded. A self-administered questionnaire provided information on demographic data such as height, weight and body mass index (BMI), number of years of training and frequency of training sessions/sports activities per week. The subjects were split into three race categories: short distance (3–5 miles), medium distance (10–13 miles), and long distance (26+ miles) running. The short distance race was a 4 mile trail in Lock Haven, PA, mostly on grass with occasional hills. The half marathons were mostly street running with occasional rolling hills. The long distance running included 50 miles single-loop, mostly unpaved course (82% dirt, 18% paved) in Rothrock State Forest that included uphill, level and downhill sections. Demographic data of the subject participants is depicted in Table 1.

**Table 1:** Demographic and training profile data.

Distance group	Short	Medium	Long
Running days/week	5.6 ± 0.9	6 ± 1	6 ± 0
Running miles/week	35 ± 7	40 ± 15	60 ± 0
Age (years)	20 ± 2	25 ± 6	23 ± 3
Gender	7 Male, 2 Female	6 Male, 4 Female	2 Male, 1 Female
Height (m)	1.73 ± 0.05	1.70 ± 0.10	1.73 ± 0.03
Weight (Kg)	54.43 ± 13.15	64.86 ± 7.71	67.13 ± 3.63
Body Mass Index (Kg/m^**2**^**)**	19.4 ± 4.5	23.0 ± 1.8	23.6 ± 0.9
Experience in marathon running (years)	8 ± 3	9 ± 7	10 ± 7

**Figure 1:**
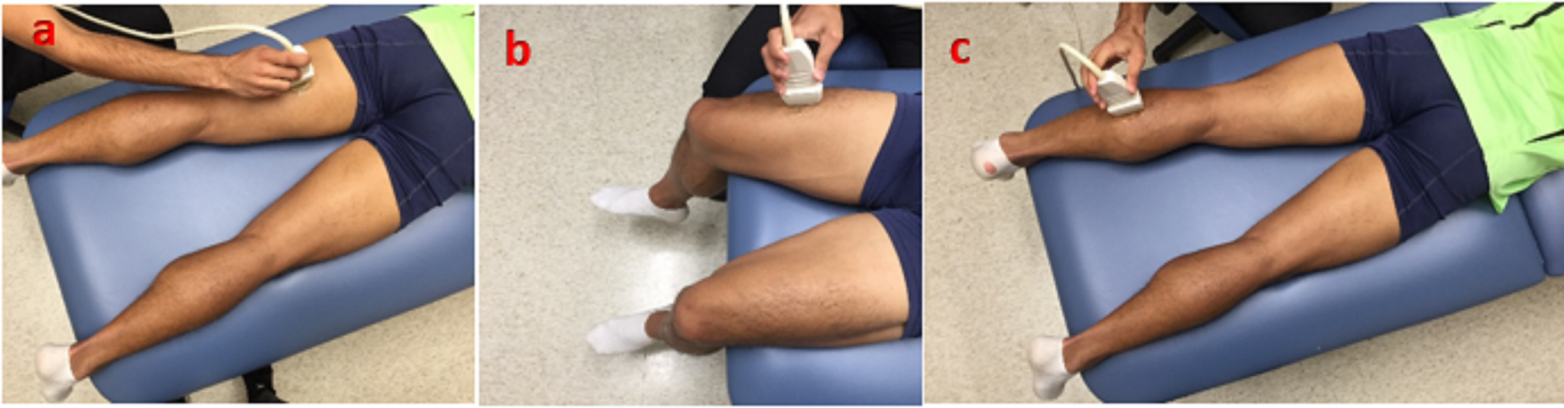
Ultrasound imaging with the transducer at different postures. (A) Posterior thigh muscles (B) anterior thigh muscles (C) calf muscles.

Shear wave speed measurements were taken using SWE on the rectus femoris (RF), vastus lateralis (VL), vastus medialis (VM), soleus, lateral gastrocnemius (LG), medial gastrocnemius (MG), biceps femoris (BF), and semitendinosus (ST) muscles of both legs (Dubois et al., 2015). For measurement on RF, VL and VM at passively stretched posture, the subjects were asked to lie supine on the table with the calves and the knees hanging off the table. For measurement on soleus, LG, MG, BF and ST, subjects were asked to lie prone on the table with the feet off the table (Fig. 1). This position stretched the posterior muscles of the thigh while also leaving the subject in a comfortable position. For the medium and long distance races, measurements were taken at three time points: 24 h before the race, 24 h after the race, and one a week after the race. For the short distance races, only two measurements were taken (24 h before the race and 24 h after the race) because of the inability to ensure that results seen one week after the race were not affected by training of equal or greater distance during the post-race week. A pre-competition scan was performed in order to obtain a baseline level for changes in muscle mechanical properties. The hand-held transducer was placed on subjects with a light amount of contact pressure. The transducer was placed in a longitudinal orientation along the muscle until the clear image of the fibers inside muscle could be identified. Marks were placed on the skin to minimize placement differences of the probe between day measurements.

During SWE measurements, the investigator monitored the real time B-mode ultrasound images to ensure that there was no noticeable body movement. Once the transducer was kept stationary for a few seconds and muscles were clearly identified in the B-mode image, the elastography mode was then activated to measure the shear modulus of the muscle. All measurements were taken five times at each muscle for each subject by an individual examiner. The median value was used in the analyses as it is not strongly affected by skewed or outlier values.

For the statistical analysis, a linear regression analysis was performed between right and left leg measurements to determine possible correlations between changes of shear modulus in both limbs. In order to measure how close the data is to the fitted regression line, the coefficient of determination was used. Secondly, a linear mixed model with running distance and recovery time as fixed-effects followed by post hoc Bonferroni correction was applied on each muscle using IBM SPSS statistics software (IBM, SPSS Inc., Chicago, IL, USA). The normality of data distribution was analyzed by the Shapiro–Wilks test. For evaluating the homogeneity of variances on all muscles, Levene’s test was used to assess the degree of variance for all of the muscles measurements. For all analyses, significance was accepted at *p* < 0.05.

## Results

**Figure 2:**
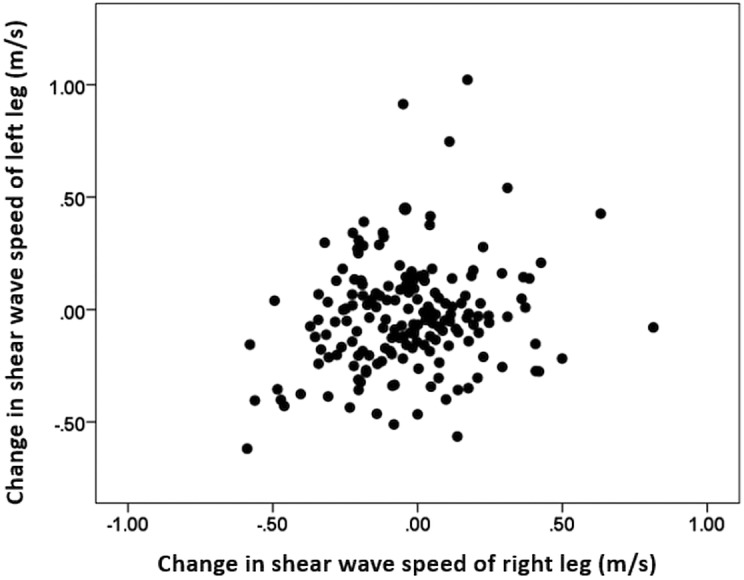
Comparison between change of the shear wave speed after competition of the right and left leg muscles.

An initial analysis of the collected data was performed to determine whether the shear wave speed of contralateral muscles from the same individual could be considered independent measurements. A very low coefficient of determination (*r*^2^ = 0.03) was obtained from the correlation of shear wave speed from the right and left leg muscles (Fig. 2), suggesting that the bilateral change in the muscle shear modulus is weakly related, and other factors may have stronger effects on these measurements. Therefore, data obtained on each leg was considered as an independent sample (separate subject) for statistical analysis. Table 2 shows the average shear wave velocity ±  standard deviation of values before and after competition for each running distance.

From the normality test, it was observed that the data for all muscles except ST (*p* = 0.17) were not normally distributed. From the Levene’s test, it was found that the error variance of the shear modulus for all muscles except ST (*p* = 0.01) is equal across groups of recovery time and running distances, meaning that more cautious interpretation is needed for the data obtained on ST muscle.

**Figure 3:**
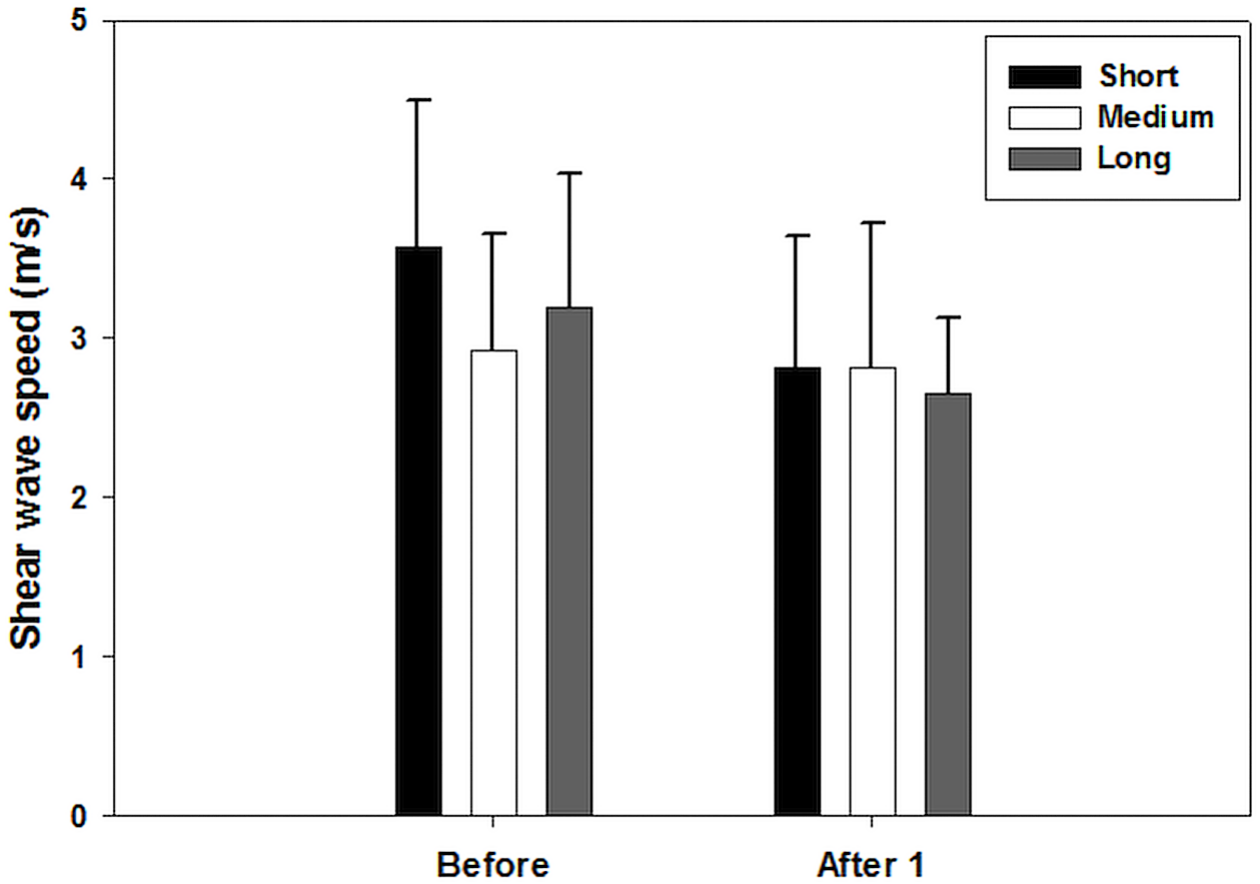
Shear wave speeds through the soleus muscle before and after competition for each distance group. (After 1 represent*s* one day after the race). This muscle shows time to have a significant effect on the average shear wave velocity values.

**Figure 4:**
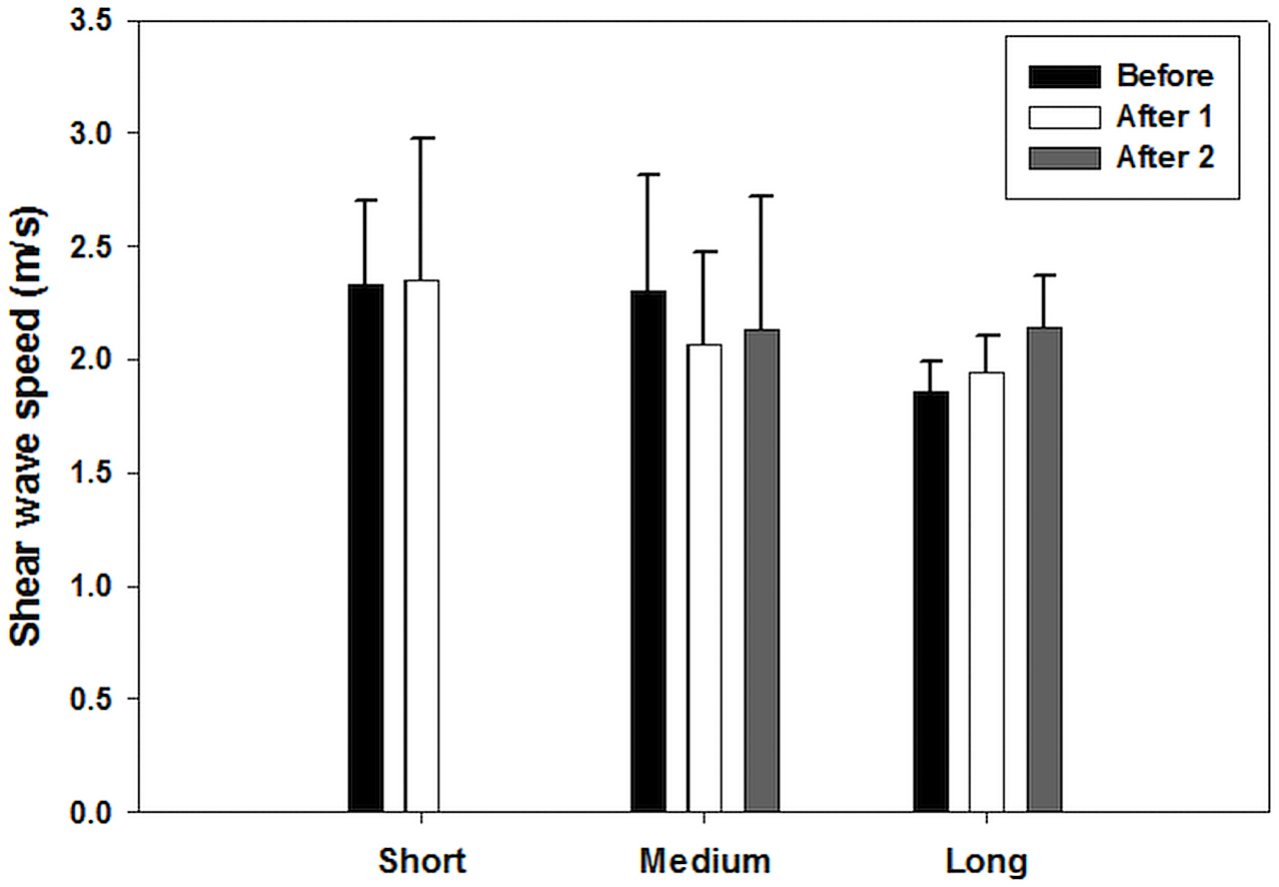
Shear wave speeds through the BF muscle at various running distances for each running time. (After 1 and After 2 represent one day and one week after the race, respectively). This muscle shows distance to have a significant effect on the average shear wave velocity values.

**Figure 5:**
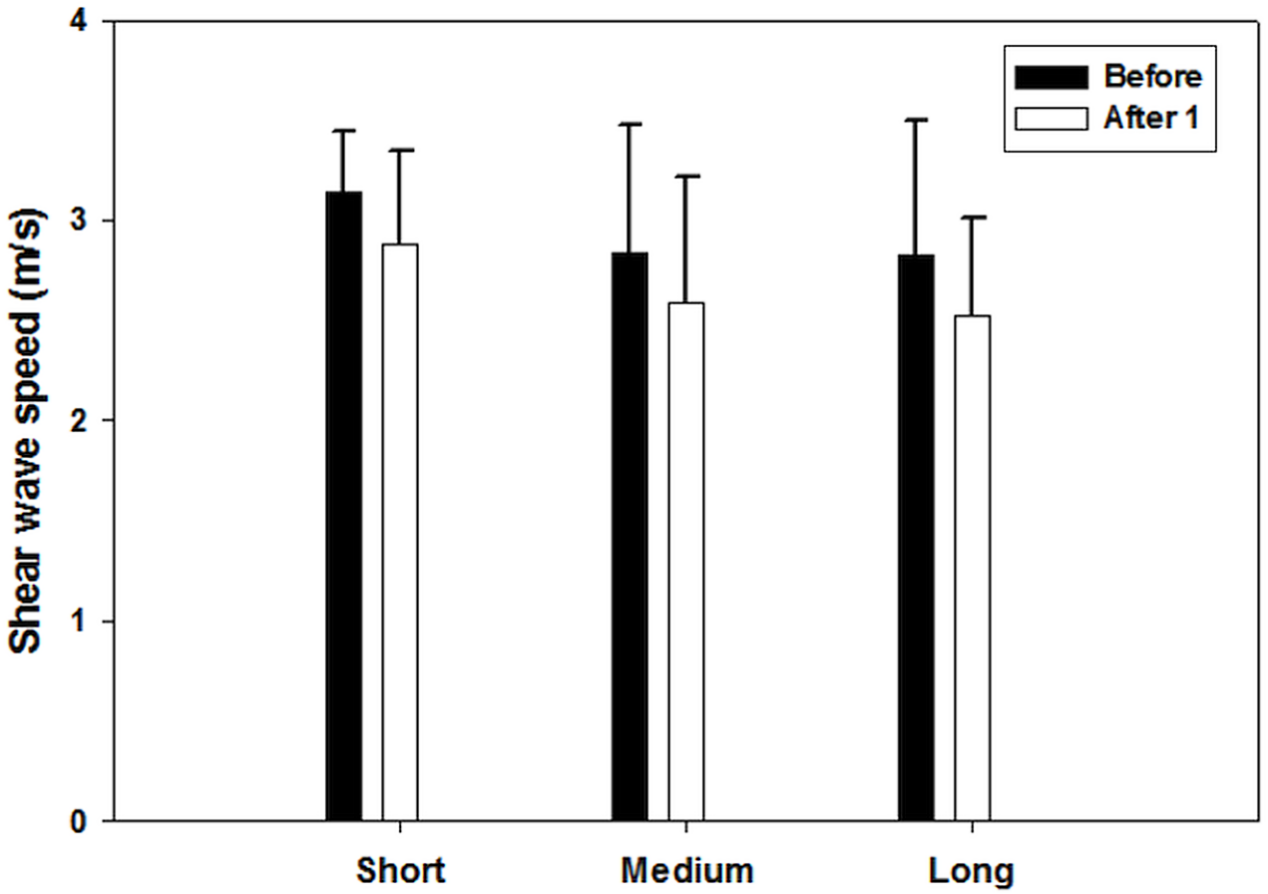
Change in shear wave speeds through the ST muscle grouped by distance and time. (After 1 represents one day after the race).

**Figure 6:**
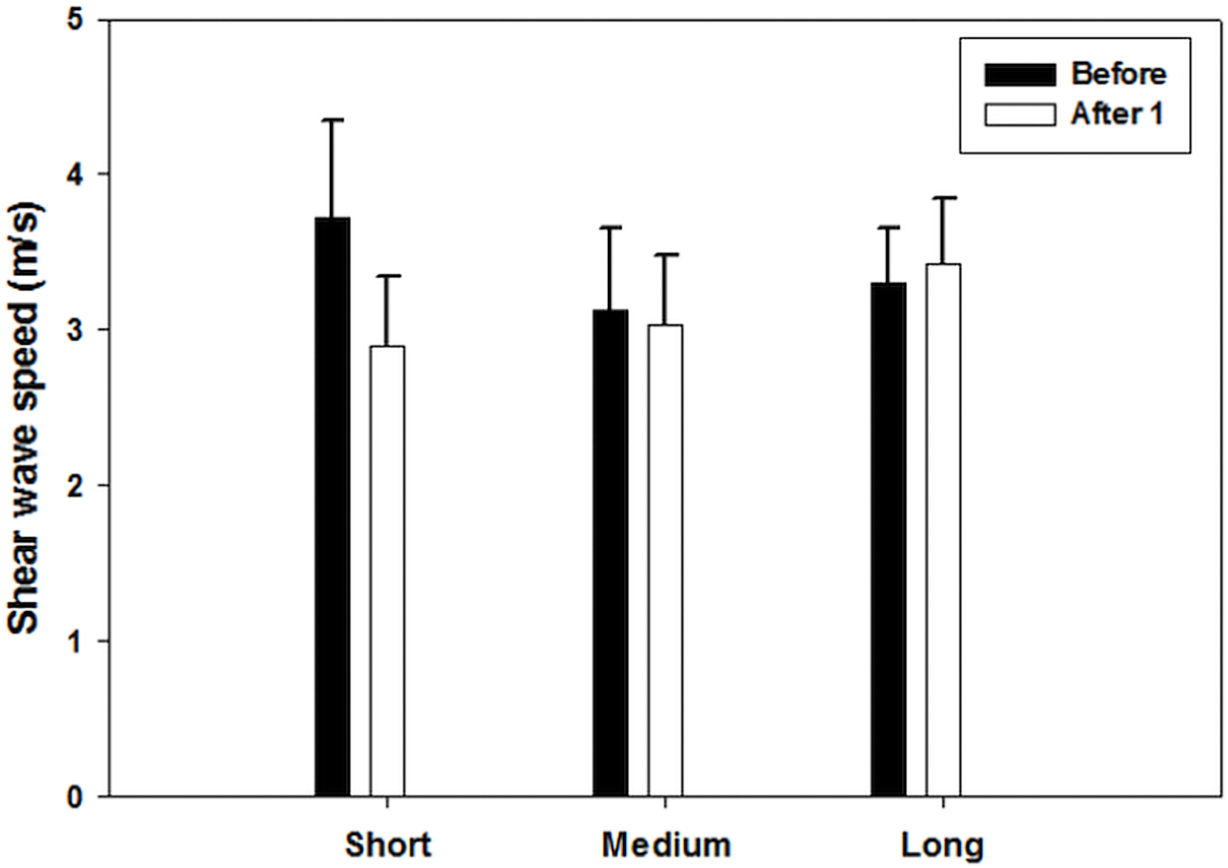
Change in shear wave speeds through the RF muscle grouped by distance and time. (After 1 represents one day after the race).

From the linear mixed effect model, it was observed that time and running distance have an effect on the mechanical properties of muscles. The results of linear mixed models including effect of fixed parameters and interactions are shown in Tables 3–7. Time was found to have a significant effect on the soleus muscle (*p* = 0.05). In the short distance group, the shear wave velocity of the soleus decreased from a mean value of 3.57 m/s before the race, to a mean value of 2.81 m/s one day after the race. Figure 3 shows the average change in shear wave speed of the soleus muscle before and after competition, separated by distance group. It should be noted that the average shear wave speed value decreased for each distance group. In addition, distance was found to have a significant effect on the BF muscle (*p* = 0.02) (Fig. 4), ST muscle (*p* = 0.02) (Fig. 5) and VL muscle (*p* < 0.01). For the short distance group, the average shear wave speed value in BF muscle appeared almost constant, while the medium and long distance groups showed an increasing and decreasing trend for shear wave speed, respectively. The RF muscle exhibited interaction between time and distance factors (*p* = 0.02), meaning that there was no similar change trend after running for all distance groups. In the short distance running group, the decreasing trend of shear wave speed was found for the RF muscle from a mean value of 3.72 m/s before the race, to a mean value of 2.90 m/s one day after the race (Fig. 6).

**Table 2:** Change in shear wave velocity (mean standard deviation) 24 hours before, 24 hours after (After 1) and one week after competition (After 2). Muscles with noticeable changes are bolded.

Shear wave velocity at different distance group (m/s)								
	Short		**Medium**			Long		
	Before	After1	Before	After1	After2	Before	After1	After2
**Soleus**	**3.57 ± 0.92**	**2.81 ± 0.83**	**2.92 ± 0.74**	**2.81 ± 0.91**	**2.59 ± 0.96**	**3.20 ± 0.83**	**2.66 ± 0.47**	**2.70 ± 0.34**
MG	2.74 ± 0.58	2.43 ± 0.38	2.44 ± 0.26	2.38 ± 0.37	2.46 ± 0.42	2.51 ± 0.35	2.64 ± 0.59	2.49 ± 0.37
LG	2.60 ± 0.55	2.45 ± 0.47	2.45 ± 0.50	2.30 ± 0.38	2.47 ± 0.32	2.46 ± 0.35	2.39 ± 0.25	2.24 ± 0.40
**BF**	**2.33 ± 0.37**	**2.35 ± 0.62**	**2.30 ± 0.51**	**2.07 ± 0.40**	**2.13 ± 0.59**	**1.86 ± 0.13**	**1.94 ± 0.16**	**2.14 ± 0.23**
**ST**	**3.14 ± 0.30**	**2.88 ± 0.47**	**2.84 ± 0.64**	**2.59 ± 0.63**	**2.47 ± 0.77**	**2.83 ± 0.67**	**2.52 ± 0.49**	**2.99 ± 0.60**
**RF**	**3.72 ± 0.62**	**2.90 ± 0.44**	**3.13 ± 0.53**	**3.03 ± 0.45**	**3.06 ± 0.84**	**3.30 ± 0.35**	**3.42 ± 0.43**	**3.18 ± 0.47**
VM	2.64 ± 0.5	2.64 ± 0.60	2.32 ± 0.55	2.45 ± 0.57	2.50 ± 0.54	2.57 ± 0.52	2.54 ± 0.87	2.55 ± 0.47
VL	2.64 ± 0.68	2.46 ± 0.43	2.58 ± 0.55	2.54 ± 0.62	2.43 ± 0.59	2.81 ± 0.34	2.88 ± 0.46	2.95 ± 0.53

**Table 3:** Linear mixed model results for BF muscle.

Source	Type III sum of squares	Degree of freedom	Mean square	*F* ratio	*P* value
Corrected model	2.51	7	0.36	1.58	0.15
Distance	1.75	2	0.88	3.86	0.02
Time	.14	2	0.07	0.30	0.74
Distance * Time	.66	3	0.22	0.97	0.41
Error	24.01	106	0.23		
Total	574.64	114			
Corrected total	26.52	113			

**Table 4:** Linear mixed model results for RF muscle.

Source	Type III sum of squares	Degree of freedom	Mean square	*F* ratio	*P* value
Corrected model	7.80	7	1.11	3.35	<0.01
Distance	1.11	2	0.55	1.66	0.20
Time	1.31	2	0.65	1.96	0.15
Distance * Time	3.32	3	1.11	3.33	0.02
Error	35.31	106	0.33		
Total	1,198.48	114			
Corrected total	43.11	113			

**Table 5:** Linear mixed model results for ST muscle.

Source	Type III sum of squares	Degree of freedom	Mean Square	*F* ratio	*P* value
Corrected model	5.95	7	0.85	2.43	0.02
Distance	2.83	2	1.42	4.05	0.02
Time	1.27	2	0.63	1.81	0.17
Distance * Time	0.10	3	0.33	0.95	0.42
Error	37.08	106	0.35		
Total	920.18	114			
Corrected total	43.03	113			

**Table 6:** Linear mixed model result for soleus muscle.

Source	Type III sum of squares	Degree of freedom	Mean square	*F* ratio	*P* value
Corrected model	11.50	7	1.64	2.33	0.03
Distance	2.15	2	1.07	1.52	0.22
Time	4.46	2	2.23	3.16	0.05
Distance * Time	2.00	3	0.67	0.95	0.42
Error	74.86	106	0.71		
Total	1,056.82	114			
Corrected total	86.35	113			

**Table 7:** Linear mixed model result for VL muscle.

Source	Type III sum of squares	Degree of freedom	Mean square	*F* ratio	*P* value
Corrected model	15.89	7	2.27	4.93	<0.01
Distance	8.73	2	4.37	9.49	<0.01
Time	0.98	2	0.49	1.06	0.35
Distance * Time	3.15	3	1.05	2.28	0.08
Error	48.78	106	.46		
Total	950.94	114			
Corrected total	64.67	113			

**Table 8:** Post hoc Bonferroni test results for shear wave speed of BF muscle between different running distances.

			95% CI		
Running distance	Mean difference	Standard error	Lower bound	Upper bound	*P* value
Short-medium	0.18	0.10	−0.07	0.42	0.24
Short-long	−0.36	0.14	−0.70	−0.03	0.03
Medium-long	−0.19	0.13	−0.5	0.12	0.44

**Table 9:** Post hoc Bonferroni test results for shear wave speed of ST muscle between different running distances.

			95% CI		
Running distance	Mean difference	Standard error	Lower bound	Upper bound	*P* value
Short-Medium	0.38	0.13	0.07	0.68	0.01
Short-Long	−0.23	0.17	−0.65	0.18	0.54
Medium-Long	0.15	0.16	−0.24	−0.53	1

**Table 10:** Post hoc Bonferroni test results for shear wave speed of VL muscle between different running distances.

			95% CI		
Running distance	Mean difference	Standard error	Lower bound	Upper bound	*P* value
Short-Medium	0.67	0.14	0.33	1.02	<0.01
Short-Long	0.32	0.20	−0.16	0.79	0.32
Medium-Long	0.36	0.18	−0.09	0.80	0.16

Additionally, from the Bonferroni post-hoc analysis conducted on individual muscles at medium and long distance running, a significant difference was obtained between shear wave speed of the BF muscle at short and long distance running (Table 8). A meaningful difference was achieved between shear wave speed of short and medium distance running for the ST and VL muscles (Tables 9 and 10). A significant difference was observed for shear wave speed of the soleus muscle before and one day after the race (Table 11). For all other muscles, there was no noticeable difference between different time points at each running distance. Overall, sixty seven percent of muscles exhibited a decreasing stiffness trend from before competition to immediately after competition, but not enough to make any assumptions based on this observation.

**Table 11:** Post hoc Bonferroni test results for shear wave speed of soleus muscle between different time points (After 1 and After 2 represent one day and one week after the race, respectively).

			95% CI		
Running distance	Mean difference	Standard error	Lower bound	Upper bound	*P* value
Before-After1	0.44	0.18	0	0.87	0.05
Before-After2	0.61	0.21	0.11	1.12	0.12
After1-After2	0.17	0.21	−0.33	0.67	1

## Discussion

This study quantified changes in mechanical properties of the thigh and lower leg muscles as a function of running distance and time after competition using SWE. The SWE was performed on healthy individuals at three race categories: short, medium and long distance running. It was found that recovery time after running has a significant effect on the soleus, RF and ST muscles, while running distance has a significant effect on the BF, RF and ST muscles. Sixty-seven percent of muscles exhibited a decreasing stiffness trend from before competition to immediately after competition, indicating that recovery time does in fact have a significant effect on the mechanical properties of muscles. These results also suggest that the effect of running distance varies between different muscles or subjects. Overall, SWE could effectively measure the mechanical properties of leg muscles before and after running races of various distances. Therefore, SWE could be a potential tool for evaluating physiological changes of muscles after running competition.

Previous SWE studies evaluating changes in muscles stiffness caused by exercise focused on one distance group at a time without isolating running distance as a crucial factor. For instance, Andonian et al. (2016) assessed changes in quadriceps stiffness before, throughout, and after an extreme mountain ultramarathon using SWE and observed a decreasing trend from before to 48 h post-race. They evaluated the shear modulus changes only in one competition distance, reporting that the stiffness values were found to be in their lowest points at mid-race. Their results are concordant with our findings for the long distance group, confirming the reduction in shear modulus of quadriceps muscles shortly after ultra-marathon running. The decrease in shear modulus observed in this study is also consistent with the results observed by Giovanelli et al. (2016) and García-Manso et al. (2011), who reported decreases in VL stiffness immediately after an uphill marathon and decreases in BF stiffness 15 min after an Ironman triathlon races, respectively, using non-invasive muscle belly deformation using tensiomyography (TMG) technique. Giovanelli et al. (2016) employed an objective protocol, in which the subjects underwent the TMG test before and immediately after the race, reporting that TMG temporal parameters decreased in all cases (−27.35% ± 18.00%, *P* < 0.01), while García-Manso et al. (2011) barely observed any changes in TMG parameters and raised some speculations based on their TMG results. Both studies analyzed their results based on the relationship developed by Pisot et al. (2008). Pisot et al. (2008) found a relationship between a decrease in muscle belly deformation and a decrease in cross-sectional muscle area. Significant negative correlations were found between the percentage change in maximum amplitude of the displacement (D_m_) and the baseline D_m_ values in BF muscle (*r* =  − 0.71, *P* < 0.01) indicating that the smaller the D_m_ value before bed rest, the larger the change induced by bed rest. The recent review paper indicated that TMG, like the SWE, has a high degree of reliability and low measurement error (Martín-Rodríguez et al., 2017). On the other hand, Green et al. (2012) reported modest increases in the shear modulus of the MG after 15 min of backwards walking (2 km/h) on an inclined treadmill, using MRE technique. Guilhem et al. (2016) reported a 28% increase in the MG shear modulus immediately after 10 sets of 30 maximal eccentric contractions of the plantar flexor muscles. However, in our study, the decrease and increase trend in BF and MG shear modulus was only observed immediately after medium and long distance running, respectively. Additionally, our mean shear modulus value and standard deviation for the BF, RF, VM, VL, ST and MG at the pre-running at the stretched postures was 5.43 ± 0.86 kPa, 13.83 ± 2.31 kPa, 6.97 ± 1.32 kPa, 6.97 ± 1.80 kPa, 9.86 ± 0.94 kPa and 7.51 ± 1.59 kPa, respectively. This is comparable to those values of 10.1 ± 2.1 kPa, 13.9 ± 3.9 kPa, 7.3 ± 0.9 kPa, 6.3 ± 1.6 kPa, 5.1 ± 1.40 kPa and 6.2 ± 1.2 kPa reported by Dubois et al. (2015). The differences between our values and the aforementioned study may be related to methodological differences between our acquisition protocol and theirs. Overall, comprehensive comparisons with other similar exercise models at similar running conditions were not possible. To our knowledge, this is the first study to provide information about the effect of running distance and recovery time on shear modulus of thigh muscles using SWE.

The change in shear modulus after running may be caused by several mechanisms. The immediate increase in shear modulus of muscles post-exercise was postulated to an increase in resting level of myoplasmic calcium caused by muscle fibers damage (Balnave, Davey & Allen, 1997; Chen et al., 2007), although the underlying mechanism of rise in muscle shear modulus remains controversial (McHugh et al., 1999). The residual number of cross-bridges between actin and myosin heads (Hill, 1968) may increase due to exercise, causing greater shear modulus (Whitehead et al., 2001). Lacourpaille et al. (2014) explored the effect of exercise induced muscle damage on muscle shear modulus and reported an increase in shear elastic modulus at longer elbow flexor muscle length (i.e., higher elbow joint angle). They attributed the shear modulus changes to the varying number of cross-bridges and amount of titin in muscles of different individuals. On the other hand, some activities such as stretching muscle tissue and mechanical agitation of a muscle were reported to break some of the residual cross-bridges spontaneously formed at rest and reduce shear modulus (Pournot et al., 2016). Eriksson Crommert et al. (2015) reported decreased MG shear modulus immediately after seven minutes of massage on relaxed leg muscles. Other explanations for changes in shear modulus of the elbow flexors muscles have been associated with shortening of parallel, non-contractile elements in muscles (Howell et al., 1985). Hence, different mechanisms may contribute to the changes in various muscles shear modulus post exercise at different running conditions.

SWE holds good promise as a valid biomarker to quantify muscle shear modulus changes in the thigh and lower leg muscles post-exercise in runners at various running conditions. The decrease in muscle shear modulus measured by SWE can be linked to an increase in maximum shear strain during mechanical loading (Loerakker et al., 2013). Shear strain causes deformation, which plays a major role in the aetiology of deep tissue injury (DTI). As a result of increase in internal shear strain during mechanical loading, the properties of muscles change, causing an increase in lipid content and decrease in muscle tone (Scelsi, 2001), which can contribute to the decrease in muscle shear modulus. Therefore, it is necessary to minimize deformation in subjects at risk of DTI by quantifying changes of muscle shear modulus post-exercise using SWE.

Our proposed protocol using SWE offers several potential application for practitioners. First, this is a localized method, which is capable of quantifying individual muscle function independent of the neighboring muscles, while other conventional methods, such as EMG has some limitations for evaluating muscles function. For example, recordings electrical activity of muscles can be contaminated by activity of other muscles and mechanical artifacts. In addition, under extreme fatigue conditions, muscles are unable to produce force, while EMGs can still receive electrical signals, meaning that its data is not reliable in all cases. Another potential application of SWE is that it may help clinicians diagnose or monitor recovery process of leg muscles noninvasively. While some stiffness may be necessary for performance, either too little or too much stiffness may cause injury. For example, higher stiffness was found to be beneficial to athletic performance of football players (Kalkhoven & Watsford, 2017). Therefore, it may be beneficial for practitioners working with athletes, particularly the ones required to perform dynamic activities such as running to consider the contribution of stiffness to athletic performance. Our proposed protocol may provide a clinical tool that can quantify the function of individual leg muscles after injury or during recovery process, which can provide better insight into leg muscles function after running competition.

There are several limitations in this study. The first limitation includes small sample size, especially for the long distance group. We acknowledge that providing more data from a larger group of people would have expanded the breadth of our study. Compression of tissue through the transducer can increase tissue stiffness due to nonlinearity of muscle tissue. Therefore, minimal compression should be applied to maintain consistent transducer contact with the skin surface. SWE measurements in muscles were subjective since the magnitude of the applied stress was controlled with operator dependent manual compression. Moreover, although muscles with various pennation angles have been studied, the influence of muscle pennation on the measurements reliability was not evaluated. Additionally, subject pre-race mileage, gender effect, recovery time, topography of training courses and intensity of training were not investigated, which may have had an influence on the variability of the data.

## Conclusions

In conclusion, SWE effectively measured the mechanical properties of leg muscles before and after running races of various distances. Recovery time had a significant effect on the soleus, RF and ST muscles, while running distance had a considerable effect on the BF, RF and ST muscles. Sixty-seven percent of muscles exhibited a decreasing shear modulus trend from before competition to immediately after competition. The results also suggest that running distance has an effect on muscle mechanical properties; however, the specific effect of time and distance seems to vary between individual muscles. Evaluating different muscles at various time points could also be extended to other sports such as baseball or powerlifting, to evaluate muscles function elicited by different types of stimulation. Overall, the preliminary results presented in this study suggest that SWE has the potential to quantify shear modulus changes of muscles under various running conditions and help provide background information needed for the design of appropriate training strategies for runners.

##  Supplemental Information

10.7717/peerj.4469/supp-1 Supplemental Information 1 Short distance running data

10.7717/peerj.4469/supp-2 Supplemental Information 2 Medium distance running data

10.7717/peerj.4469/supp-3 Supplemental Information 3 Long distance running data

## Additional Information and Declarations

### Data Availability

The following information was supplied regarding data availability: The raw data has been provided as Supplemental Files.
